# Supplementary material for: Comparative Genome Analysis of 19 Trueperella pyogenes Strains Originating from Different Animal Species Reveal a Genetically Diverse Open Pan-Genome
Source: Antibiotics (Basel). 2022 Dec 24;12(1):24. doi: 10.3390/antibiotics12010024 (PMC9854608; doi:10.3390/antibiotics12010024)
Supplement: Supplementary file 1 [file antibiotics-12-00024-s001.zip › Figure S1 (a-j) Synteny plots.pdf]

Supplementary Figure 1 (a-j) Synteny plot of investigated *T. pyogenes* complete genomes against reference *T. pyogenes* TP6375 genome.

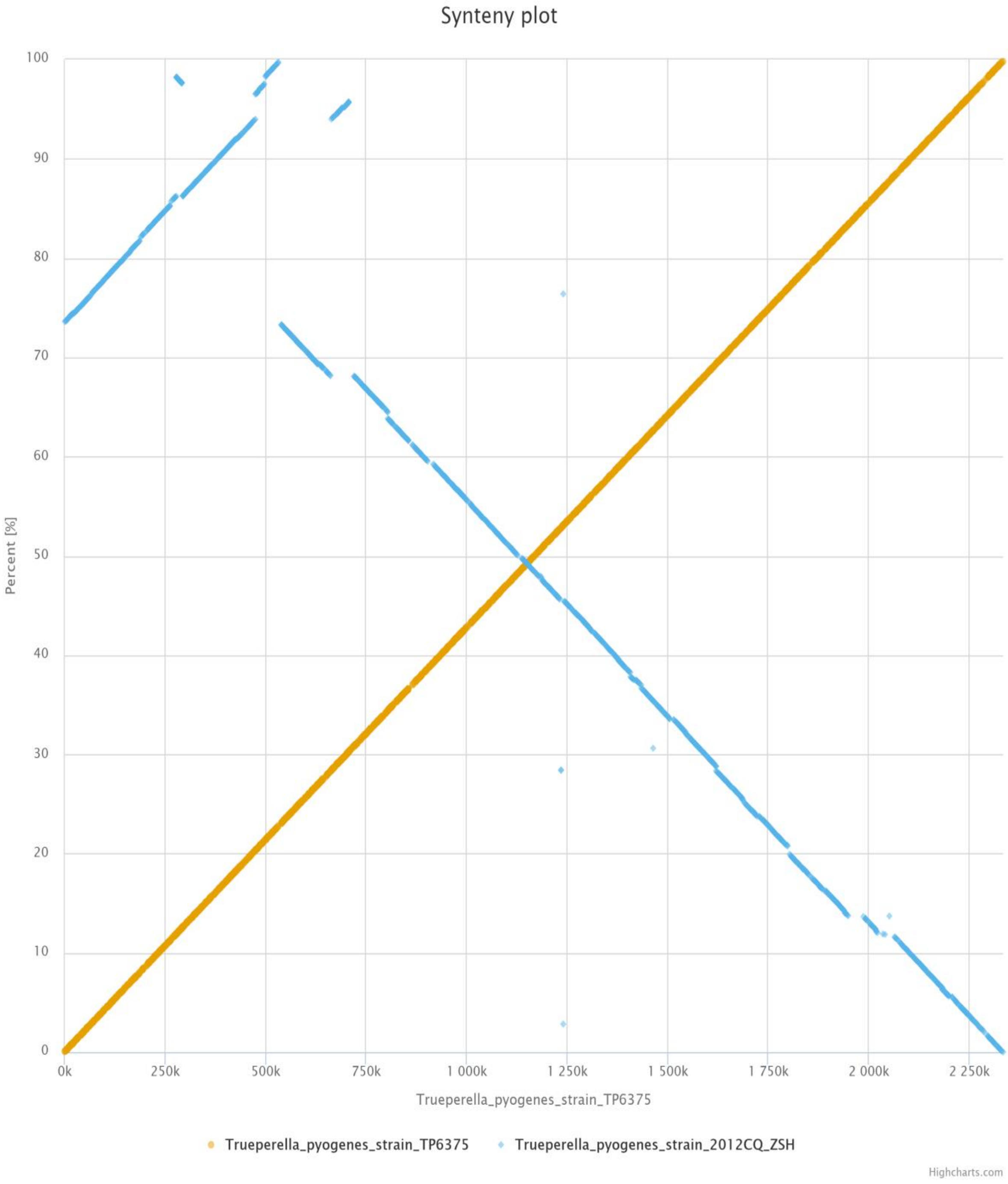

Supplementary Figure 1 (a) Synteny plot of *T. pyogenes* 2012CQ-ZSH genome against *T. pyogenes* TP6375 genome.

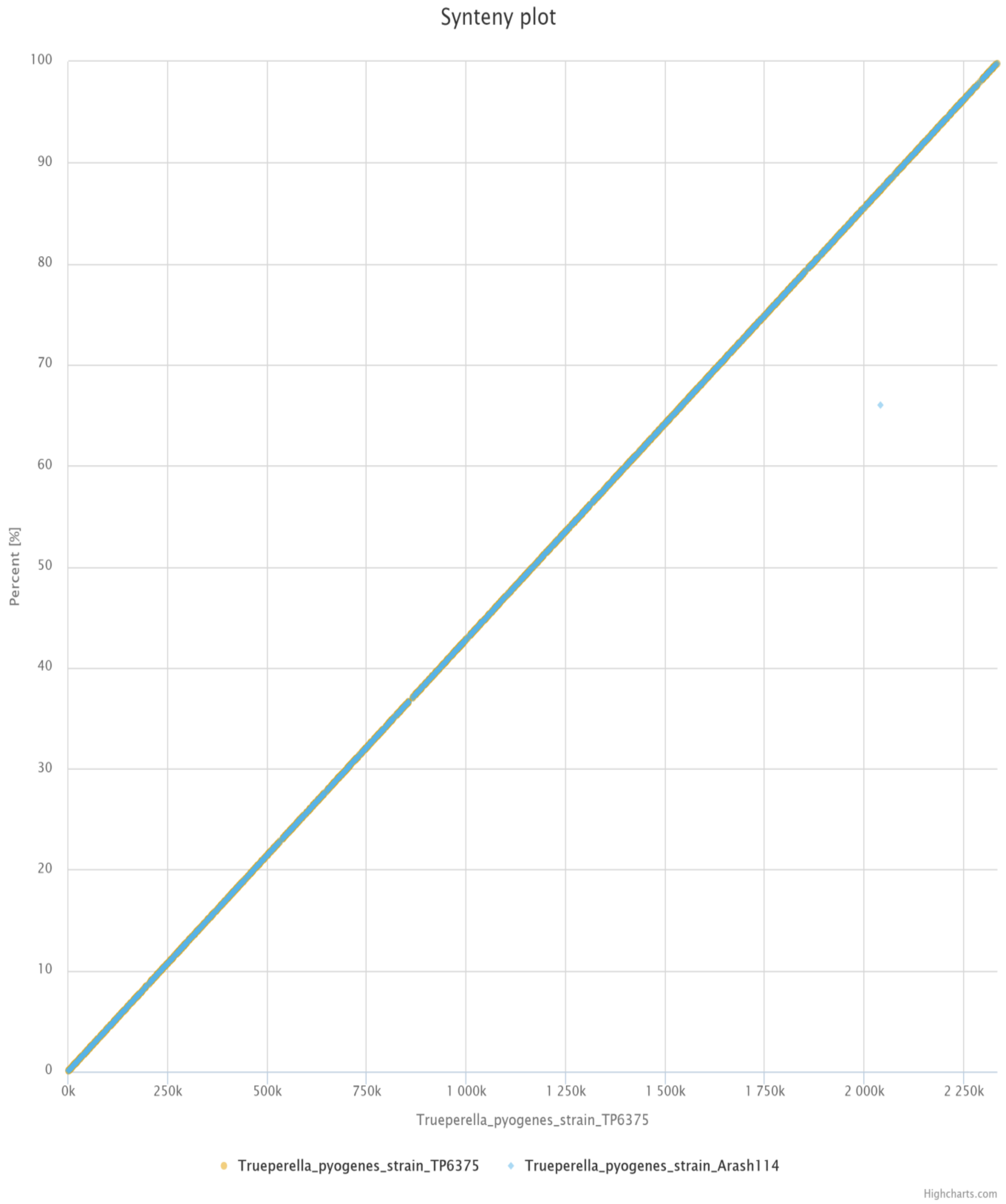

Supplementary Figure 1 (b) Synteny plot of *T. pyogenes* Arash114 genome against *T. pyogenes* TP6375 genome.

.

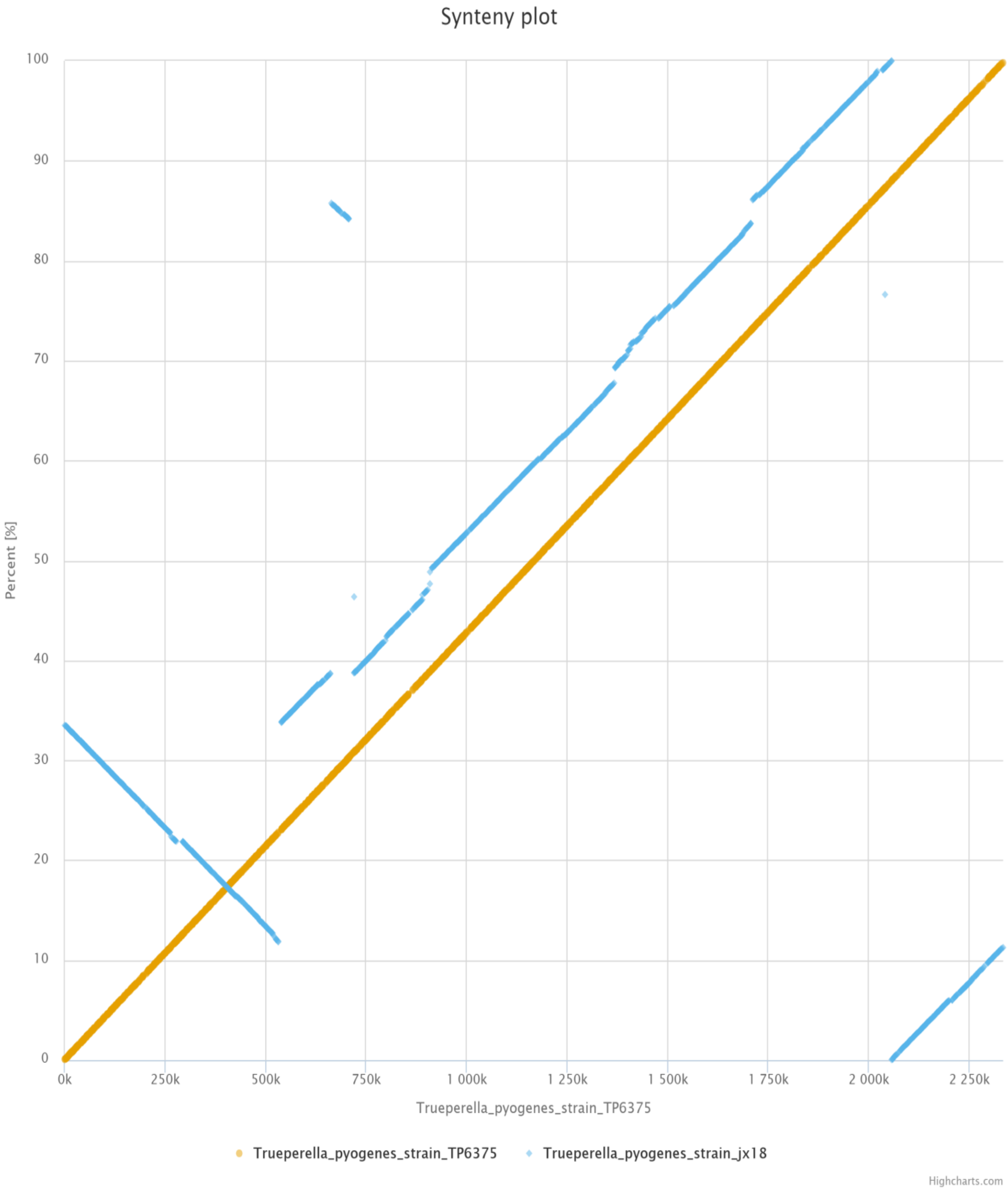

Supplementary Figure 1 (c) Synteny plot of *T. pyogenes* jx18 genome against *T. pyogenes* TP6375 genome.

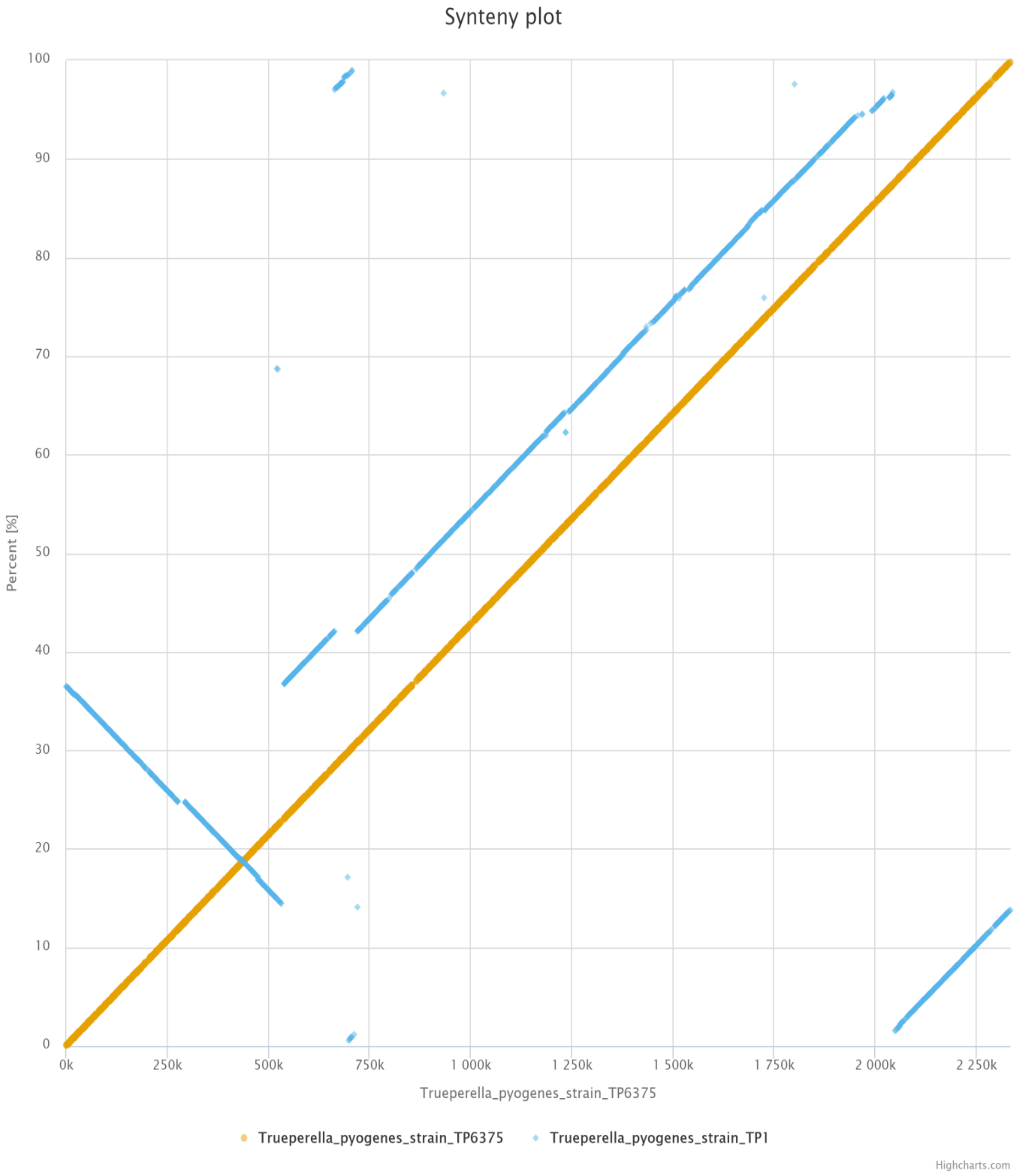

Supplementary Figure 1 (d) Synteny plot of *T. pyogenes* TP1 genome against *T. pyogenes* TP6375 genome.

.

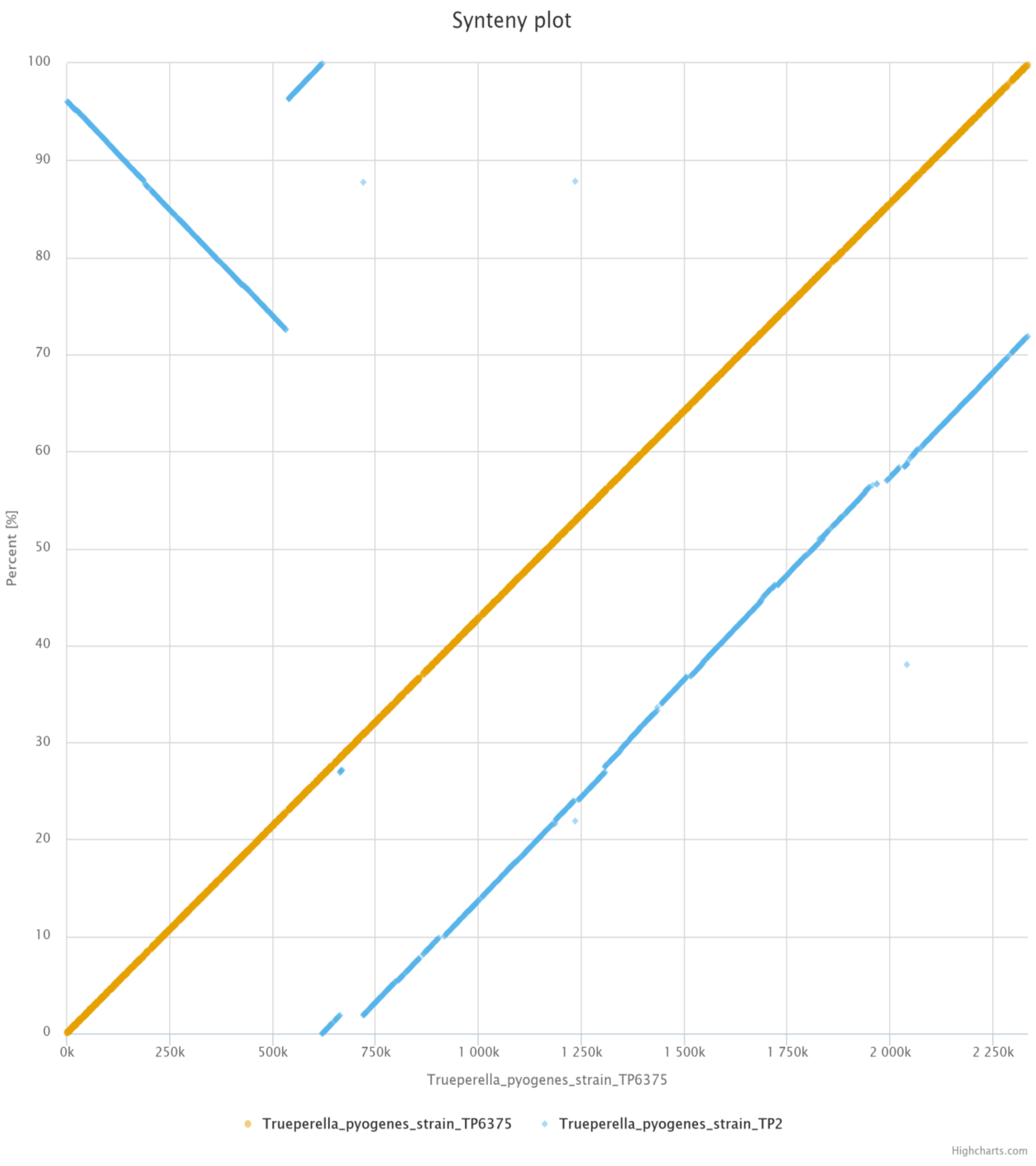

Supplementary Figure 1 (e) Synteny plot of *T. pyogenes* TP2 genome against *T. pyogenes* TP6375 genome.

.

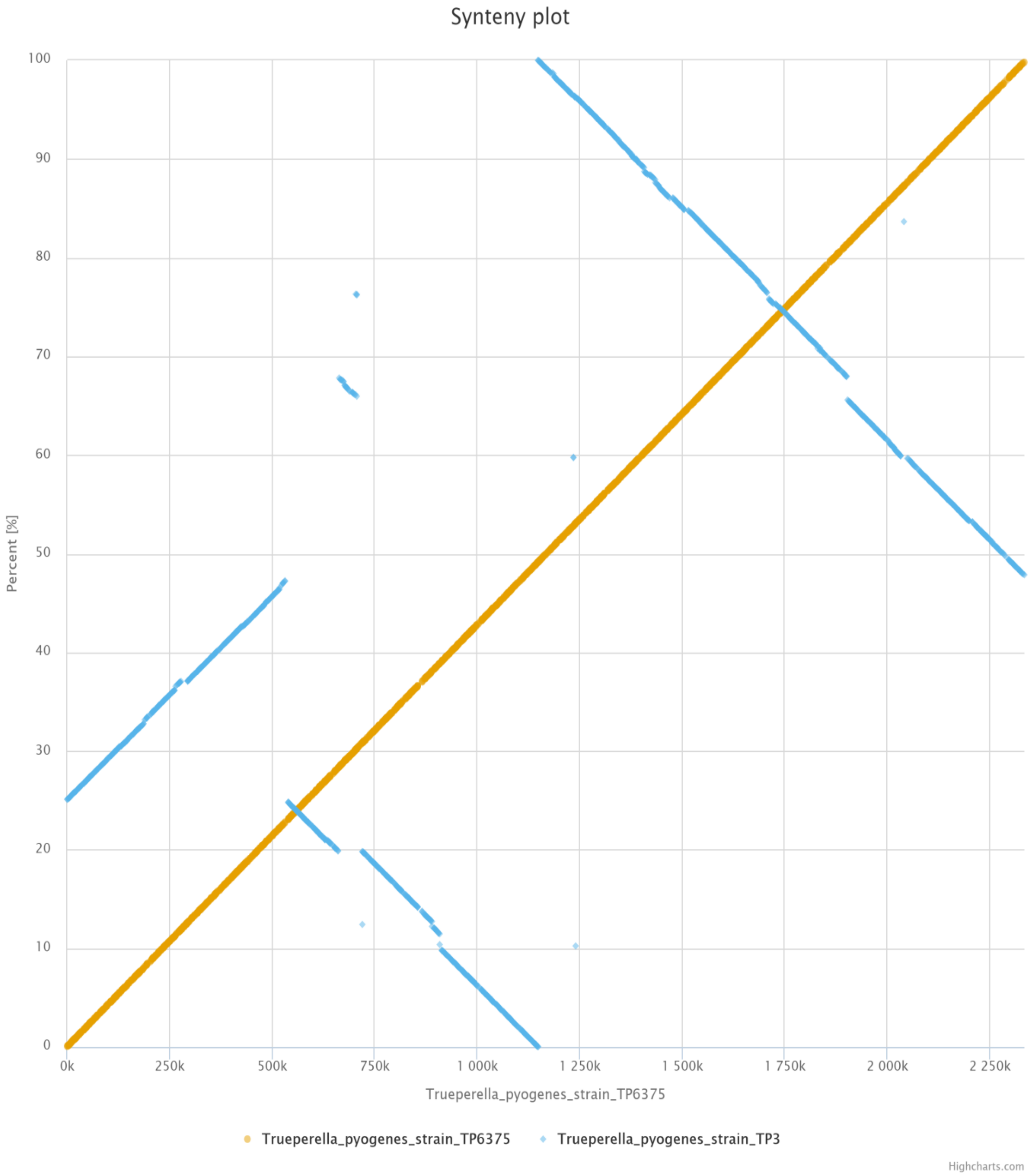

Supplementary Figure 1 (f) Synteny plot of *T. pyogenes* TP3 genome against *T. pyogenes* TP6375 genome.

.

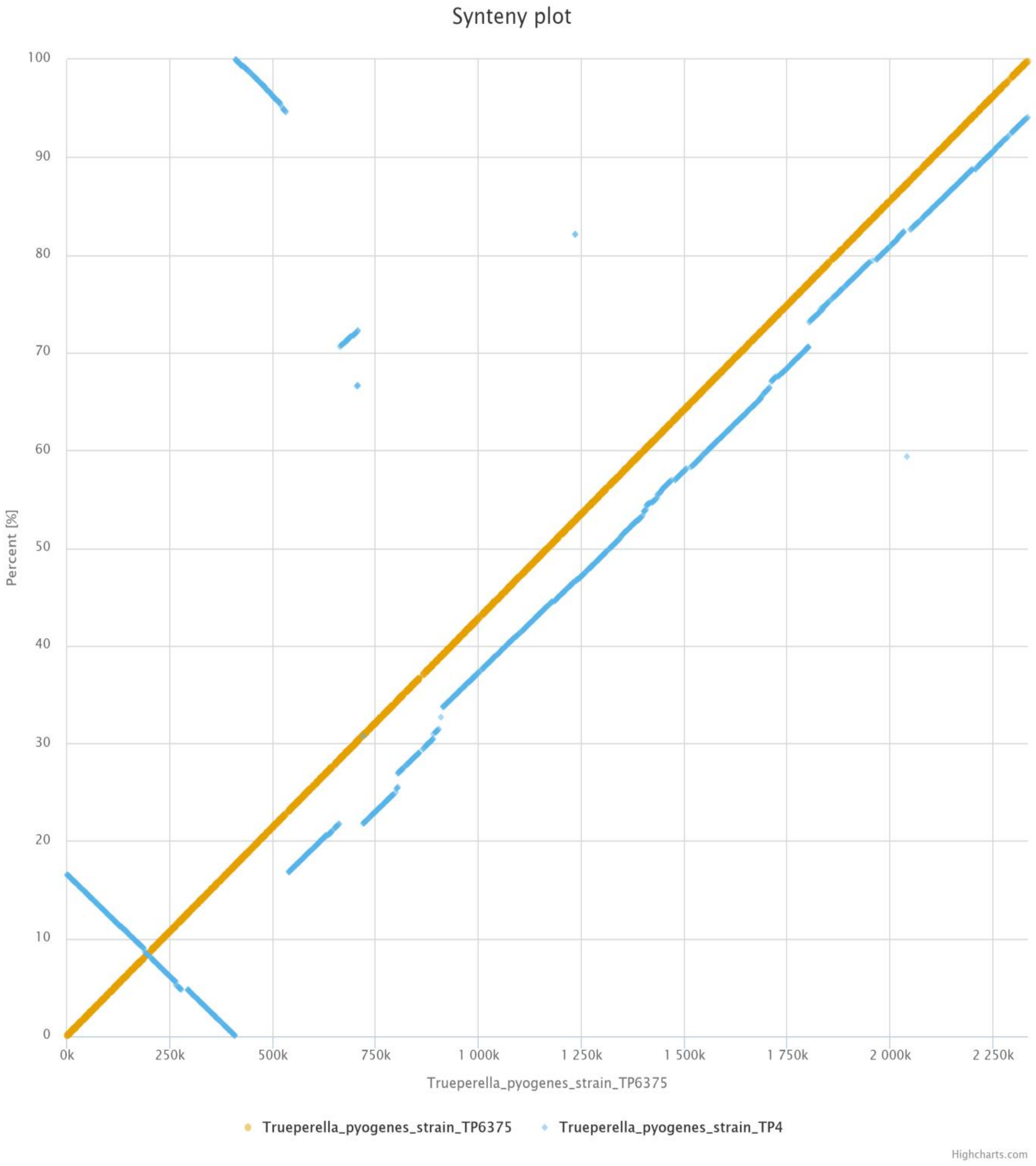

Supplementary Figure 1 (g) Synteny plot of *T. pyogenes* TP4 genome against *T. pyogenes* TP6375 genome.

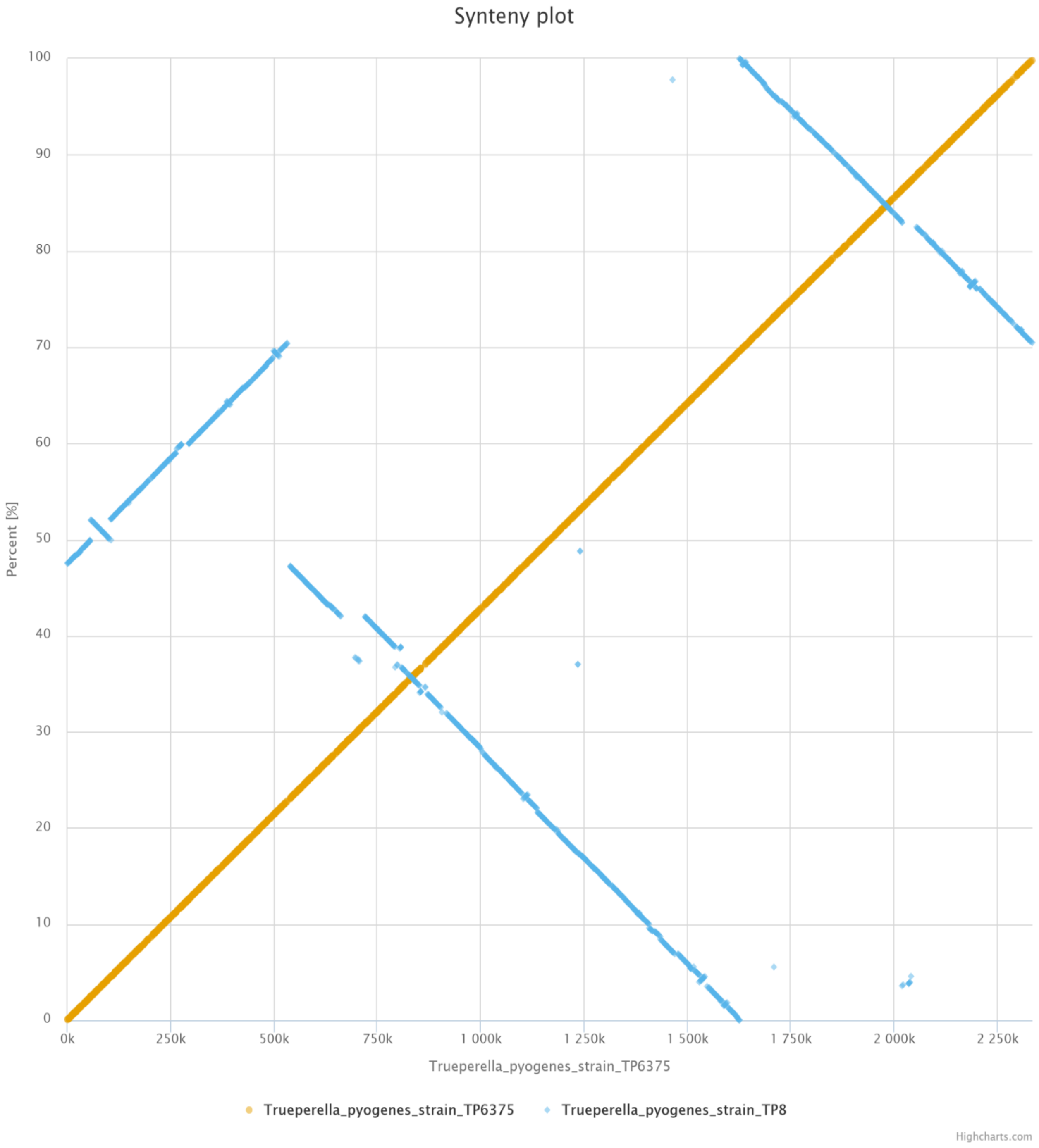

Supplementary Figure 1 (h) Synteny plot of *T. pyogenes* TP8 genome against *T. pyogenes* TP6375 genome.

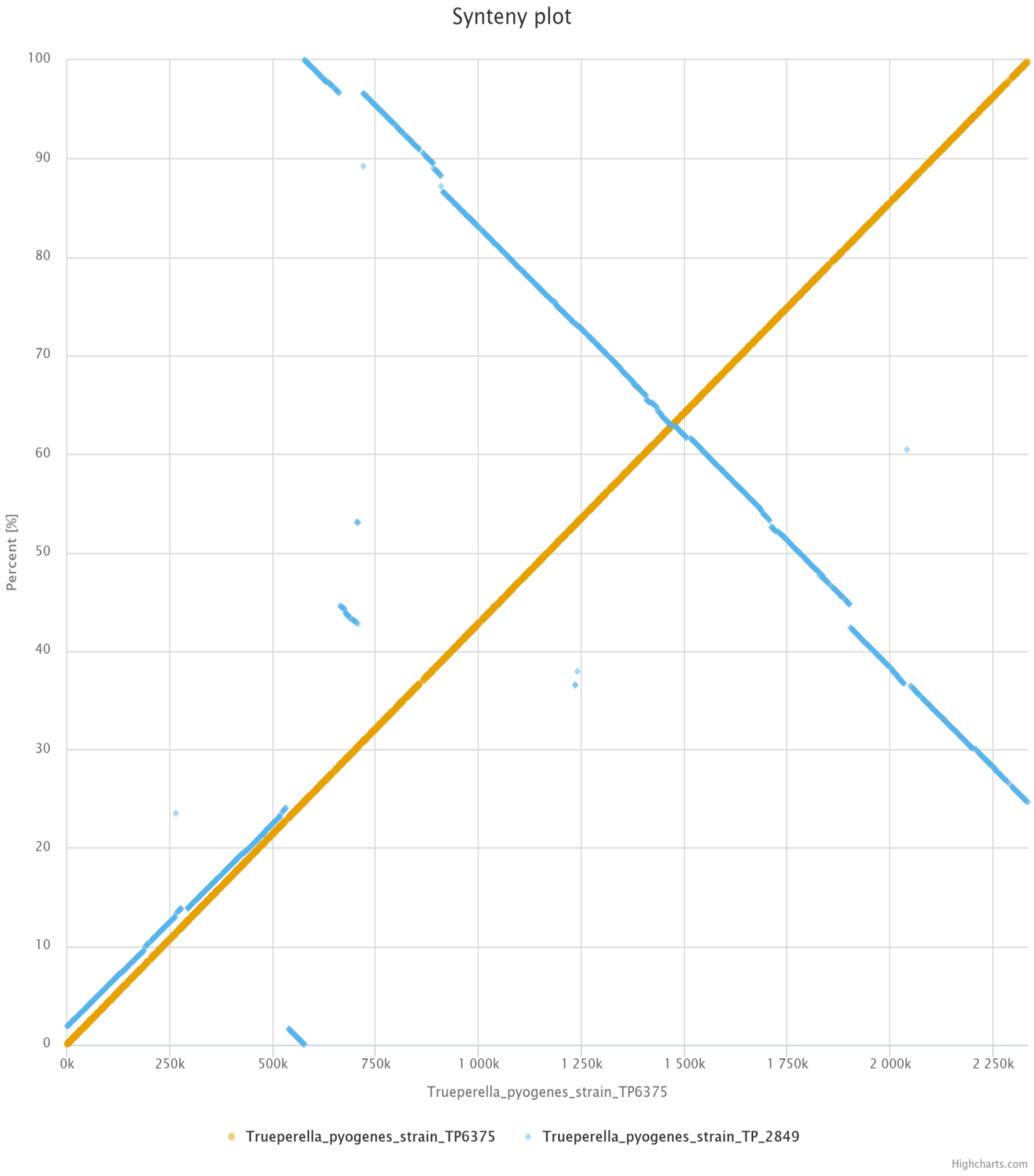

Supplementary Figure 1 (i) Synteny plot of *T. pyogenes* TP2849 genome against *T. pyogenes* TP6375 genome.

.

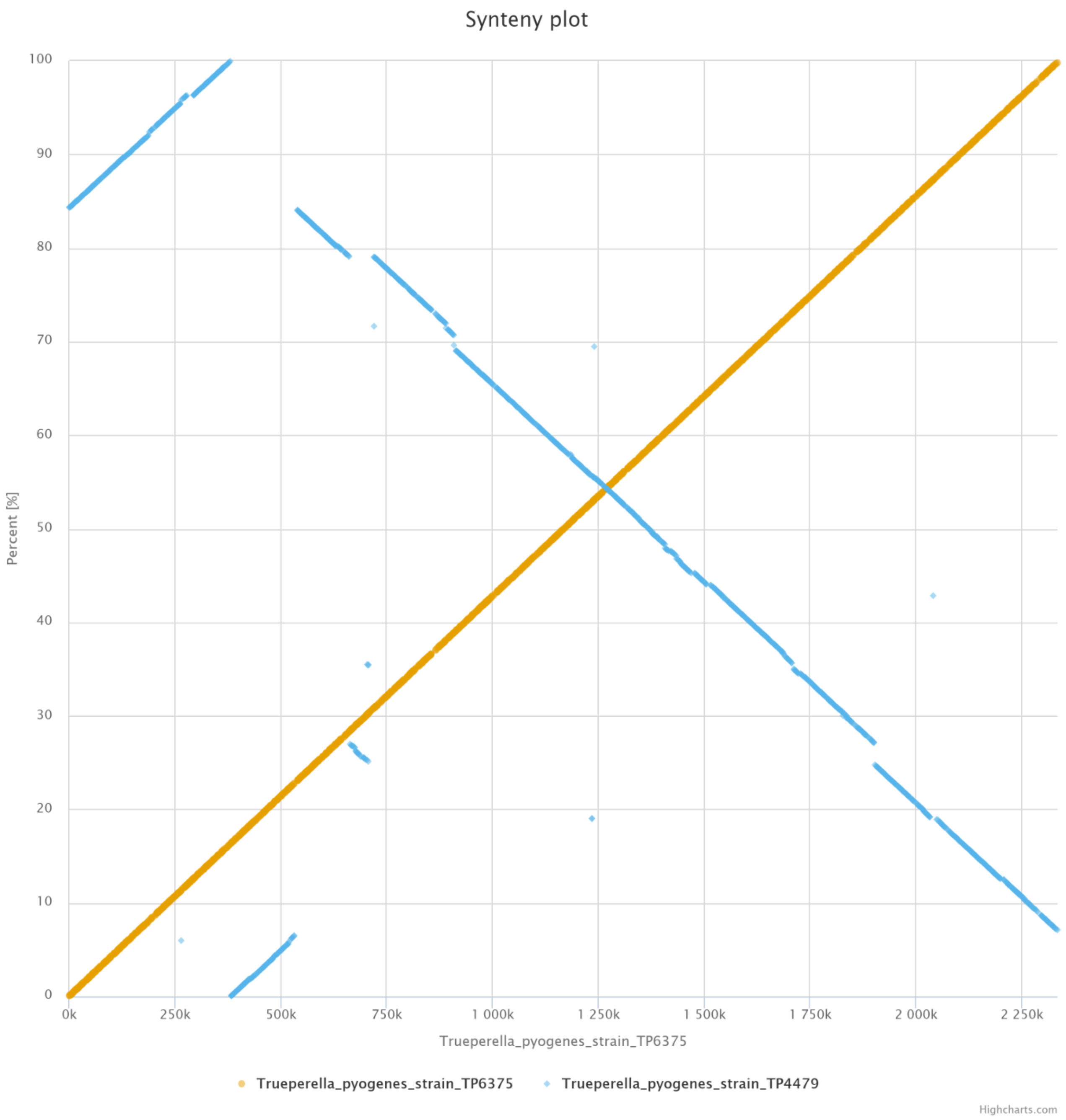

Supplementary Figure 1 (j) Synteny plot of *T. pyogenes* TP4479 genome against *T. pyogenes* TP6375 genome.

.
