# Supplementary material for: Comparative Genome Analysis of 19 Trueperella pyogenes Strains Originating from Different Animal Species Reveal a Genetically Diverse Open Pan-Genome
Source: Antibiotics (Basel). 2022 Dec 24;12(1):24. doi: 10.3390/antibiotics12010024 (PMC9854608; doi:10.3390/antibiotics12010024)
Supplement: Supplementary file 1 [file antibiotics-12-00024-s001.zip › Figure S2 (a-h) Virulence factors multisple sequence alignement.pdf]

Supplementary Figure 2 (a-h) Multiple sequence alignment of crucial virulence factors such as pyolysin (plo), collagen-binding protein A (*cbpa*), neuraminidases (*nanH* and *nanP*), and fimbriae (*fimA*, *fimC*, *fimE*, and *fimJ*) which were identified in investigated *T.pyogenes* genomesby NCBI COBALT. Only alignment positions with no gaps will be colored. Red depicts highly conserved positions and blue depicts lower conservation. This method highlights highly conserved and less conserved amino acid positions on the basis of relative entropy

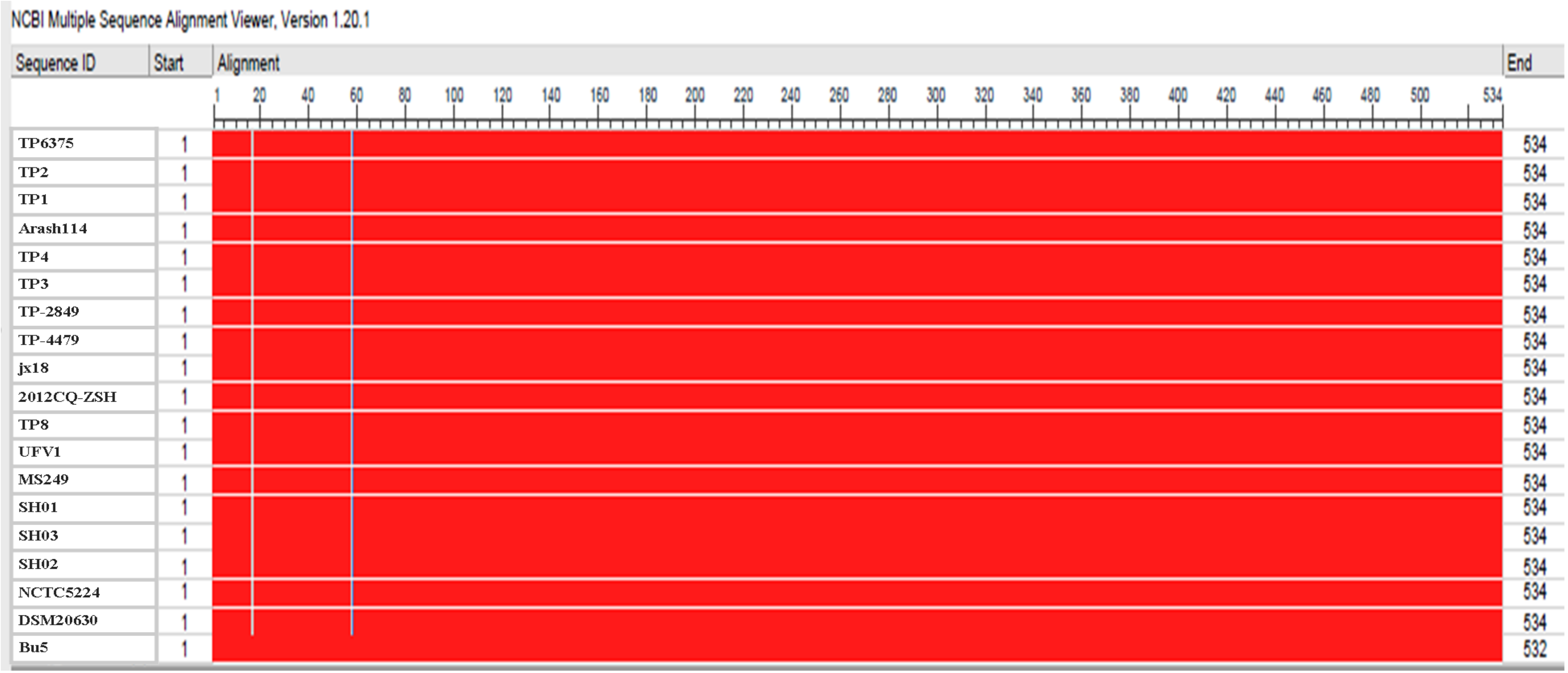

(a) Multiple sequence alignment of pyolysin (plo) proteins identified in investigated *T. pyogenes* genomesby NCBI COBALT.

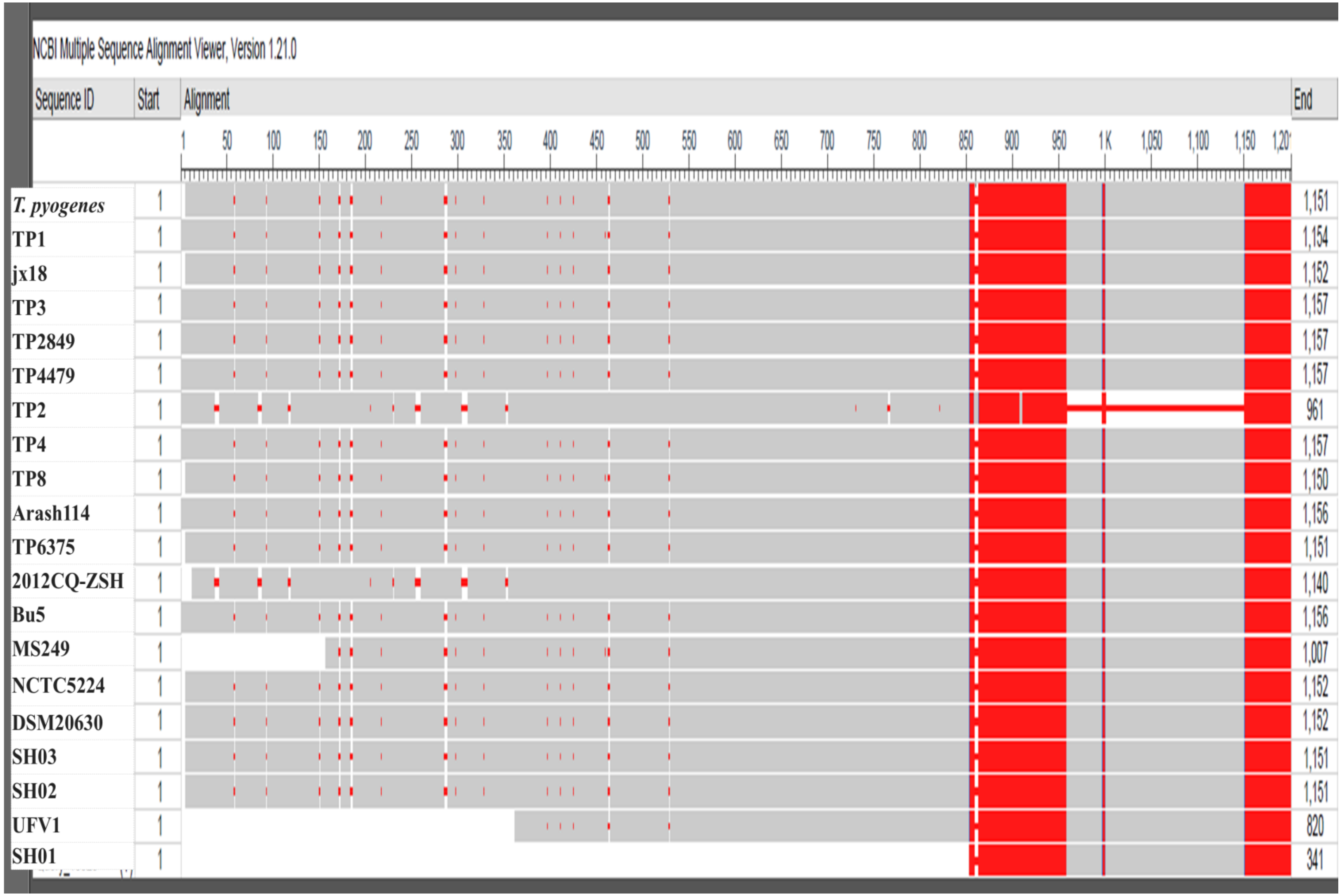

(b) Multiple sequence alignment ofcollagen-binding protein A (*cbpa*) proteins identified in investigated *T. pyogenes* genomes by NCBI COBALT.

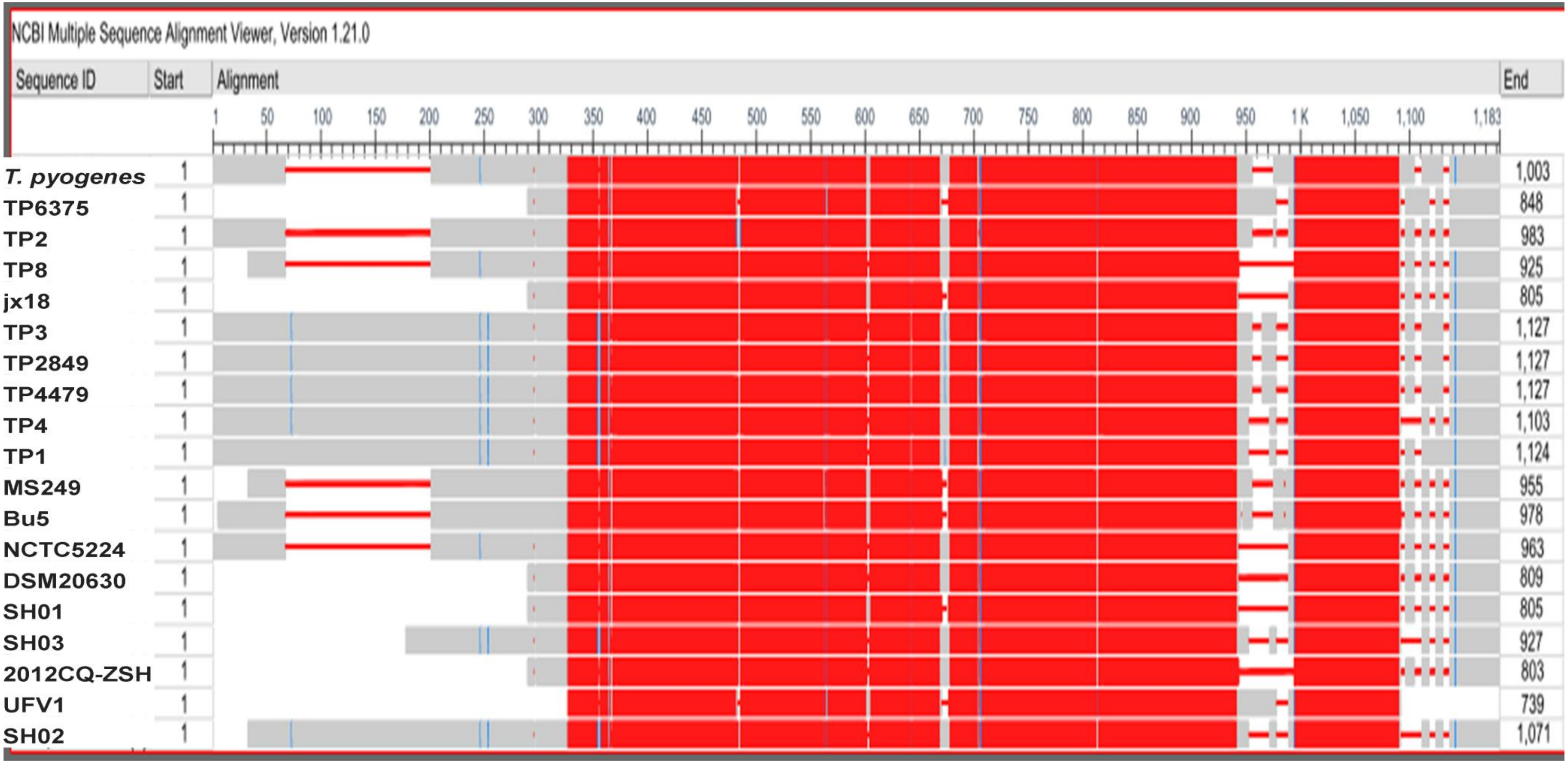

(c)Multiple sequence alignment of neuraminidases H (*nanH*)proteins identified in investigated *T. pyogenes* genomes by NCBI COBALT.

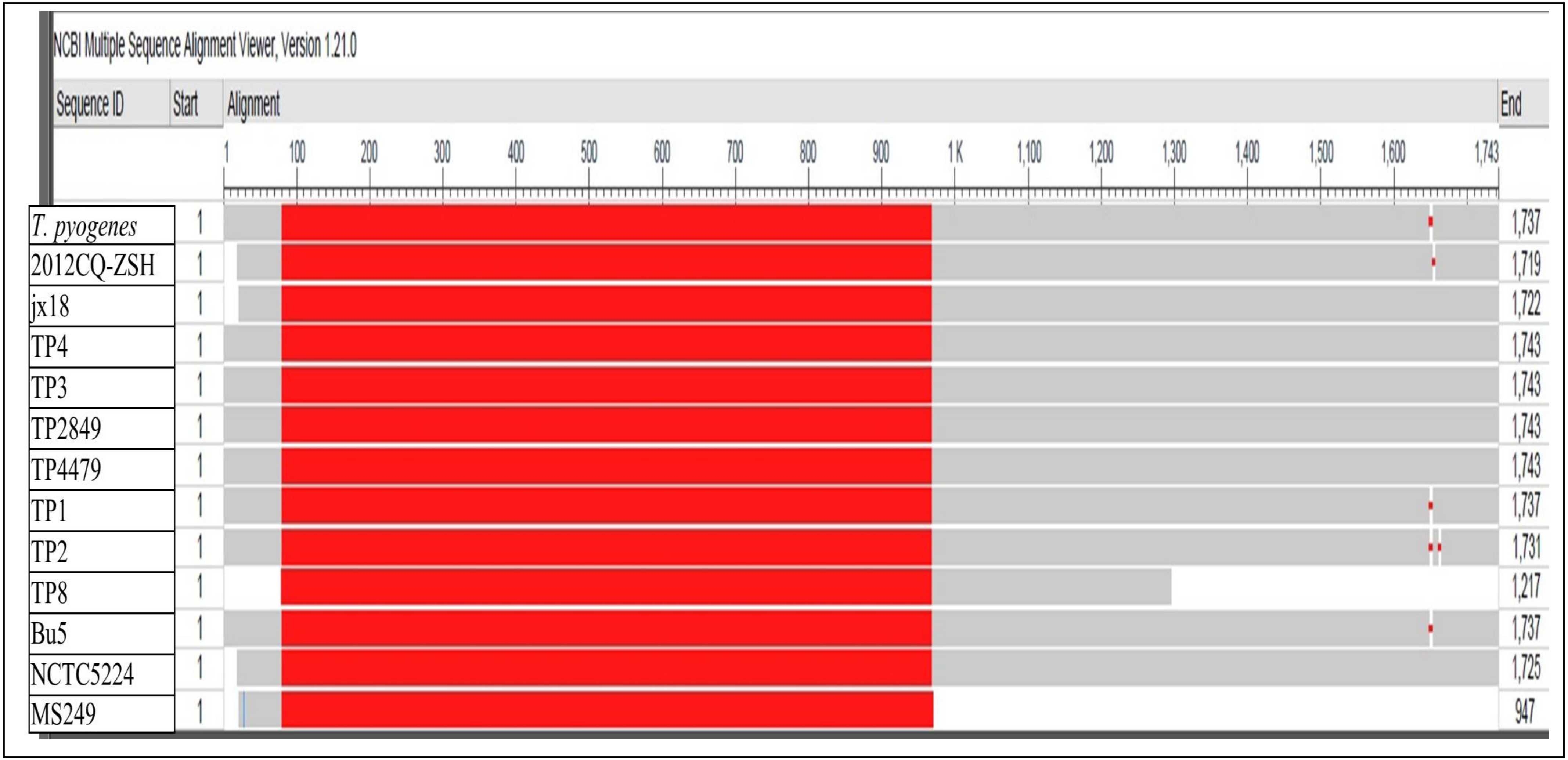

(d) Multiple sequence alignment of neuraminidases P (*nanP*) proteins identified in the investigated *T. pyogenes* genomes by NCBI COBALT.

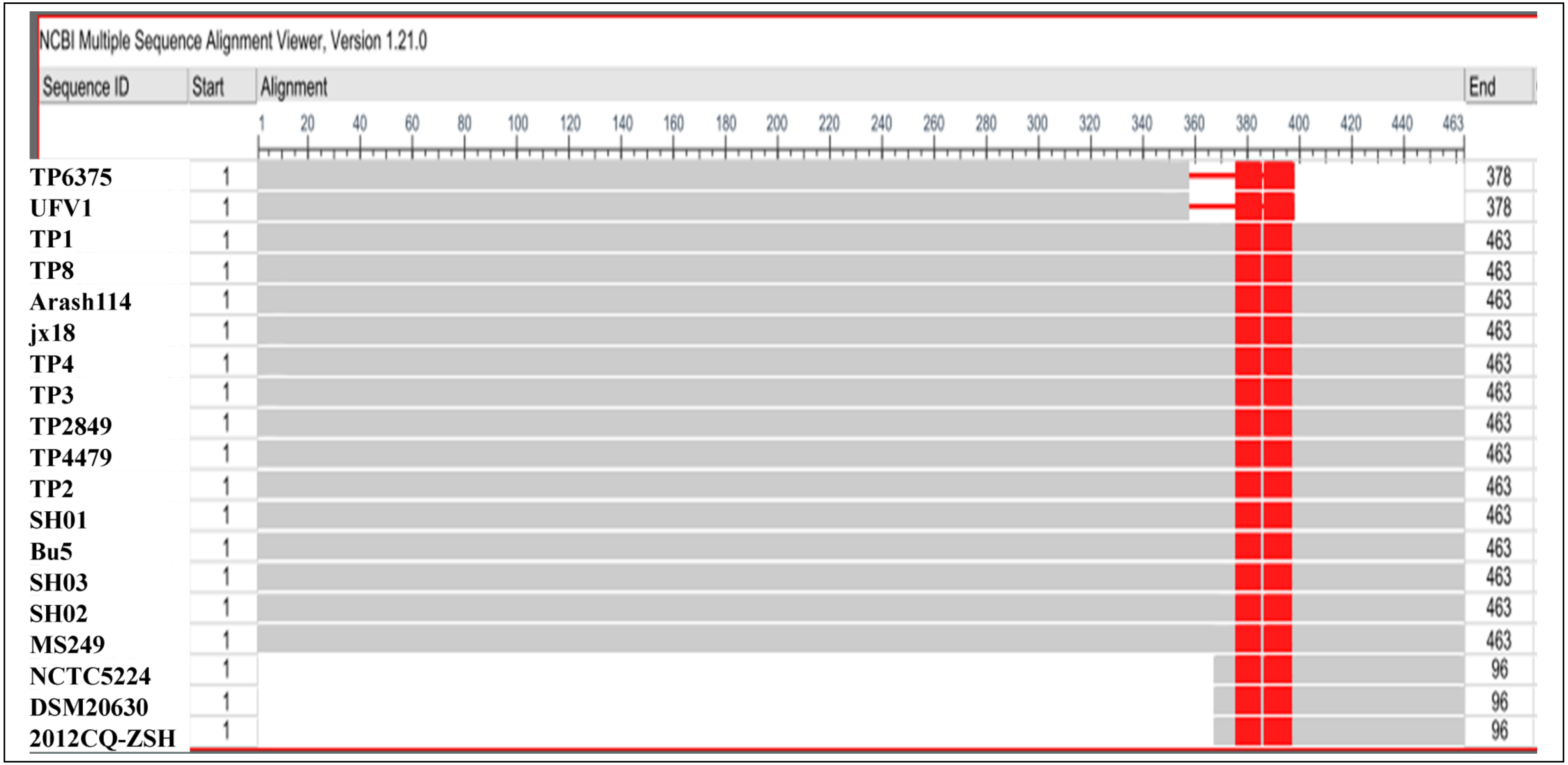

(e) Multiple sequence alignment of *fimA*proteins identified in the investigated *T. pyogenes* genomes by NCBI COBALT.

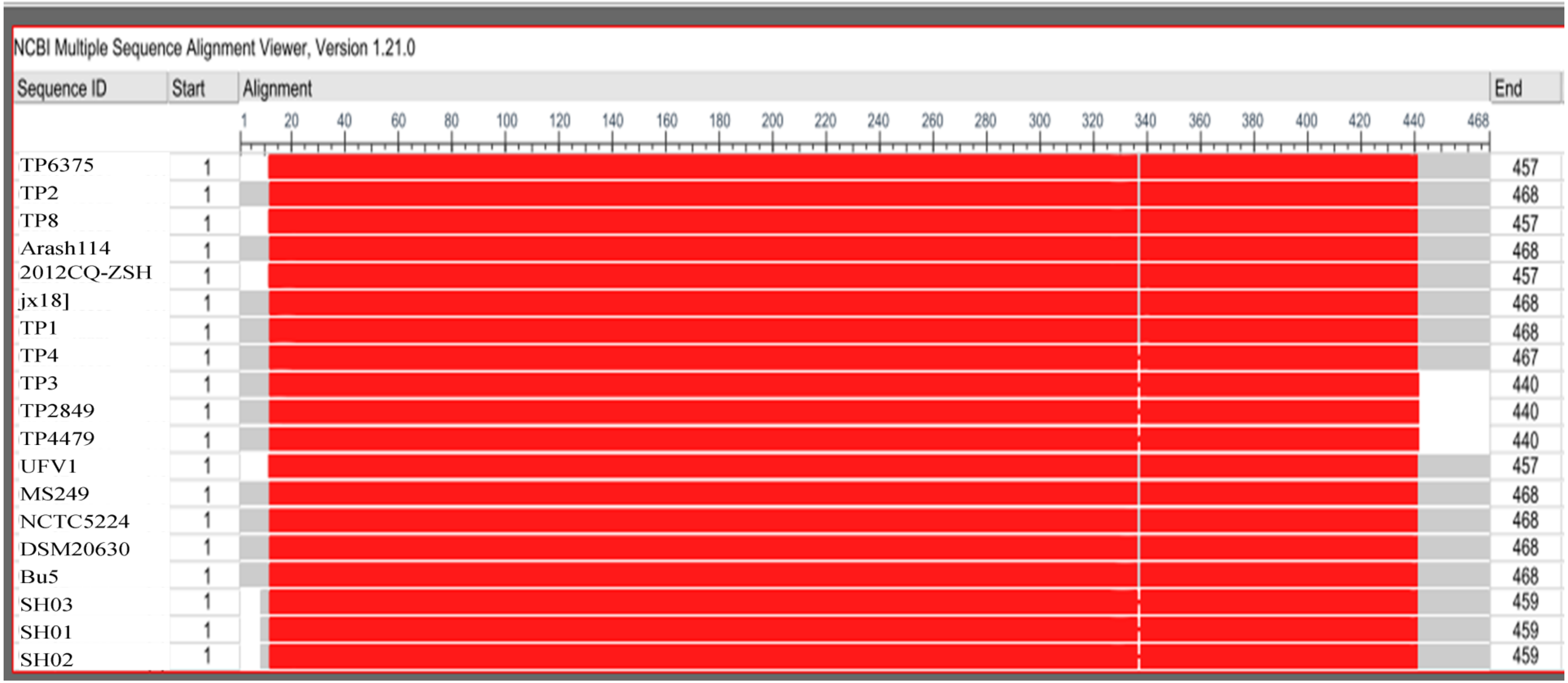

(f) Multiple sequence alignment of *fimC* proteins identified in the investigated *T. pyogenes* genomes by NCBI COBALT.

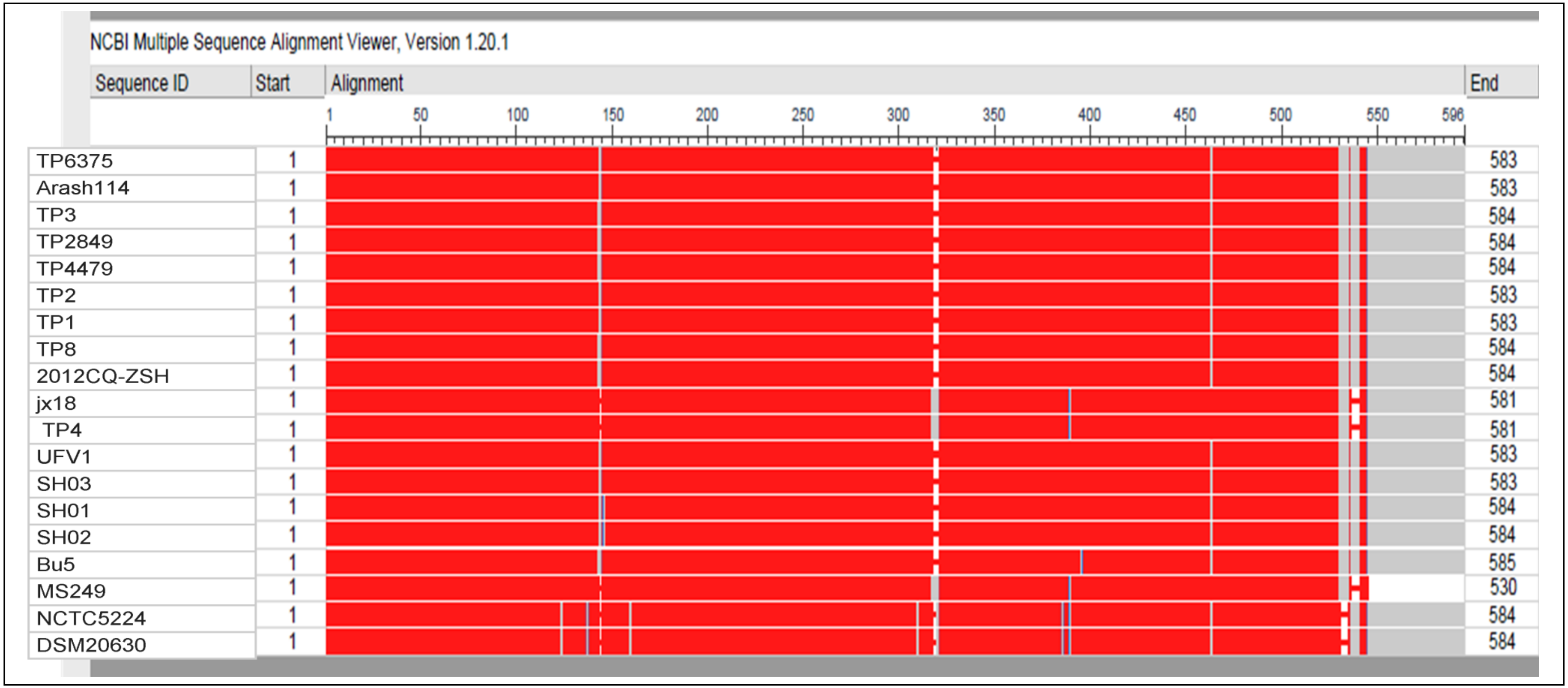

(g) Multiple sequence alignment of *fimE* proteins identified in the investigated *T. pyogenes* genomes by NCBI COBALT.

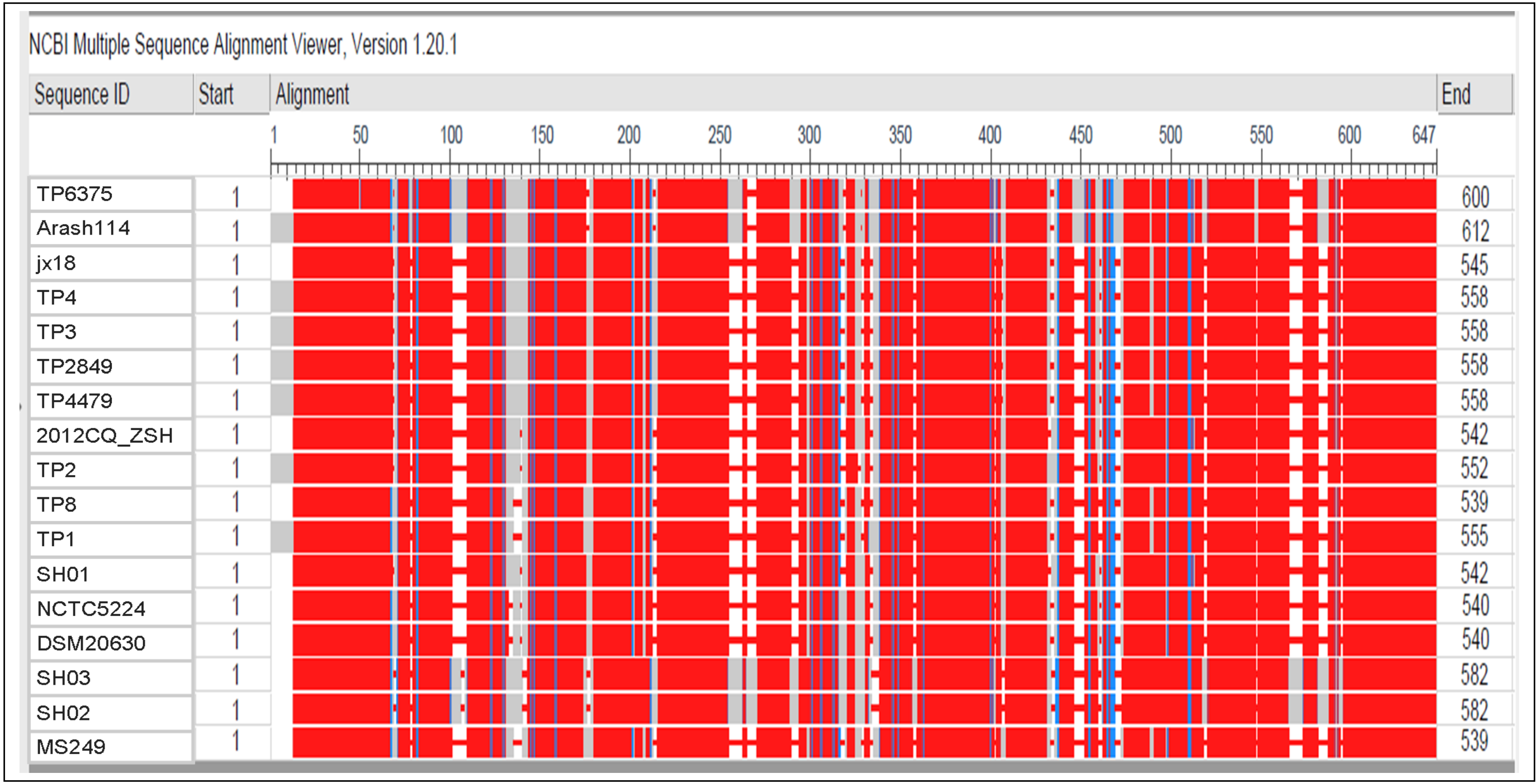

(h) Multiple sequence alignment of *fimJ* proteins identified in the investigated *T. pyogenes* genomes by NCBI COBALT.
