# Supplementary material for: Comparative Genome Analysis of 19 Trueperella pyogenes Strains Originating from Different Animal Species Reveal a Genetically Diverse Open Pan-Genome
Source: Antibiotics (Basel). 2022 Dec 24;12(1):24. doi: 10.3390/antibiotics12010024 (PMC9854608; doi:10.3390/antibiotics12010024)
Supplement: Supplementary file 1 [file antibiotics-12-00024-s001.zip › Table S1.pdf]

S1 Table. The Pan-genome and core genome development projections for investigated nineteen *T. pyogenes* strains.

**Pan-genome development extrapolations**		**Core genome development extrapolations**	
**Contigs**	**Mean number of genes**	**Contigs**	**Mean number of genes**
**2**	2258.020	**2**	1866.175
**3**	2379.335	**3**	1794.530
**4**	2480.355	**4**	1751.895
**5**	2556.745	**5**	1727.975
**6**	2626.260	**6**	1696.270
**7**	2691.545	**7**	1688.715
**8**	2755.005	**8**	1652.275
**9**	2810.825	**9**	1647.690
**10**	2866.105	**10**	1634.280
**11**	2903.440	**11**	1611.755
**12**	2951.120	**12**	1601.465
**13**	2996.825	**13**	1590.550
**14**	3035.105	**14**	1575.790
**15**	3074.840	**15**	1567.640
**16**	3109.275	**16**	1558.030
**17**	3141.885	**17**	1545.250
**18**	3180.080	**18**	1533.075
**19**	3215.565	**19**	1520.560
**Heap's Law extrapolation**		**Heap's Law extrapolation**	
Growth exponent y:<br>0.162 (alpha 0.838)		Converges to:<br>1488.954	
Confidence interval (95%) for y:<br>0.157 to 0.167		Confidence interval (95%):<br>from 1457.440 to 1520.468	
Fitted model:<br>1980.179 * x^0.162		Fitted model:<br>451.953 * exp(-x/8.271) + 1488.954	
Lower confidence interval formula:<br>1956.250 * x^0.157		Lower confidence interval formula:<br>427.939 * exp(-x/6.530) + 1457.440	
Upper confidence interval formula:<br>2004.109 * x^0.167		Upper confidence interval formula:<br>475.966 * exp(-x/10.013) + 1520.468	
