# Supplementary figures and images for: Comparative Genome Analysis of 19 Trueperella pyogenes Strains Originating from Different Animal Species Reveal a Genetically Diverse Open Pan-Genome
Source: Antibiotics (Basel). 2022 Dec 24;12(1):24. doi: 10.3390/antibiotics12010024 (PMC9854608; doi:10.3390/antibiotics12010024)

Figure S3. ANI\_MEAN300.

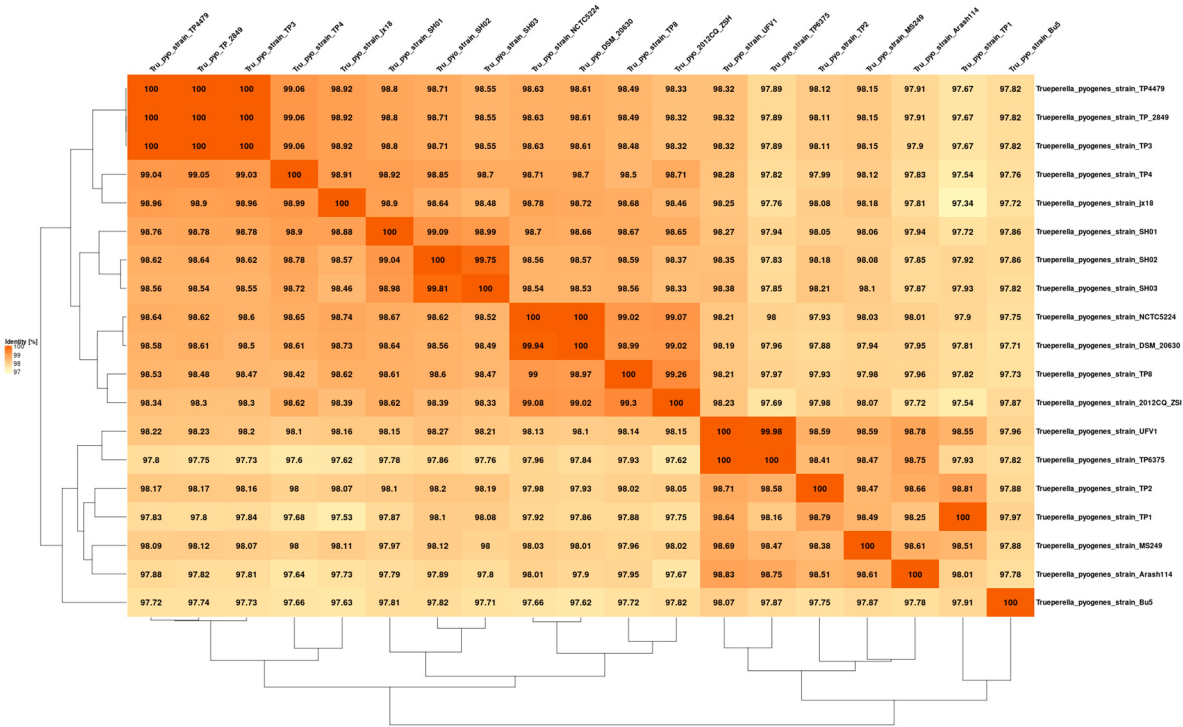

Supplement: Supplementary file 1 [file antibiotics-12-00024-s001.zip › Figure S3 ANI_MEAN300.pdf]
